# The Henry Phipps Institute for the Study, Prevention and Treatment of Tuberculosis

**Published:** 1910-09

**Authors:** 


### The Henry Phipps Institute for the Study, Prevention and Treatment of Tuberculosis.

Mr. Henry Phipps, of New York, has selected the University of Pennsylvania to carry on the work of the Phipps Institute. Mr. Phipps has already acquired ground in Philadelphia on which will be erected a hospital for this purpose. The extent of the benefaction exceeds $500,000.

The report of the committee appointed to consider the future policy of the institute has been approved by Mr. Phipps and the Trustees of the University.

The work will be divided into three general departments, each of which will be presided over by a director. For the Directorship of the Laboratory, Dr. Paul Lewis now of the Rockefeller Institute has been selected. For Directorship of the Sociological Department, Mr. Alexander M. Wilson, of the Boston Association for the Relief and Control of Tuberculosis. Dr. H. R. M. Landis has accepted the appointment as Director of the Clinical Department.

In addition to a board of eight directors who will be directly responsible to the Trustees of the University, an Advisory Council has been created and will meet annually at the Institute. The following have accepted the invitation to serve as members of this body: Dr. Samuel G. Dixon, Harrisburg, Pa.; Dr. S. McC. Lindsay, New York City; Dr. William H. Baldwin, Washington, D. C.; Dr. Hermann M. Biggs, New York City; Dr. William H. Welch, Baltimore, Md.; Dr. Theobald Smith, Boston, Mass.; Dr. Gideon Wells, Chicago, Ill.; Dr. Simon Flexner, New York City; Dr. James A. Miller, New York City; Dr. Lawrence Brown, Saranac, N. Y.; Dr. Henry Baird Favell, Chicago, Ill., and Dr. James Pratt, Boston, Mass.

